# Micronutrient requirements for stem cell transplantation patients > 100 days after transplant and during graft versus host disease: a systematic review

**DOI:** 10.1007/s00520-025-10024-8

**Published:** 2025-11-07

**Authors:** Christine Johnson, Dannielle McCormack, Teresa Brown, Helen L MacLaughlin, Claire Blake, Rachel Willims, Sarah Andersen

**Affiliations:** 1 School of Exercise and Nutrition Sciences, Queensland University of Technology, Brisbane, QLD Australia; 2 Dietetics & Food Services, Nutrition Research Collaborative, Royal Brisbane & Women’s Hospital, Brisbane, QLD Australia; 3 School of Human Movement & Nutrition Sciences, University of Queensland, Brisbane, QLD Australia

**Keywords:** Stem cell transplant, Systematic review, Micronutrient deficiency, Micronutrient supplementation, Vitamin and mineral supplement

## Abstract

**Purpose:**

There are no clinical guidelines for micronutrient supplementation and monitoring during the post-acute phase of Stem cell transplantation (SCT) and during graft versus host disease (GVHD). Hence this comprehensive systematic review aimed to evaluate the evidence for vitamin and mineral supplementation and monitoring to inform clinical practice.

**Methods:**

Eligible studies included adults who had undergone SCT more than 100 days ago or were experiencing GVHD and were prescribed a micronutrient supplement and/or had micronutrient levels monitored. Human studies published in English were retrieved from five databases (Embase, Medline, Scopus, Web of Science, and CINAHL) on the 24th of May 2024. The risk of bias and certainty of evidence were assessed through the Academy of Nutrition and Dietetics Quality Criteria Checklist and The Grading of Recommendations, Assessment, Development and Evaluation (GRADE) system. No meta-analysis was completed as part of this systematic review.

**Results:**

Sixteen studies (n = 1573) met eligibility criteria for inclusion. Micronutrients examined by these studies were vitamin D, calcium, and vitamin A. All studies had a neutral risk of bias. Studies reported variable prevalence of vitamin D deficiency, with mixed results regarding the association between vitamin D or calcium and the outcomes of reduced bone mineral density (BMD), chronic GVHD, mortality, sarcopenia, or fractures. The GRADE certainty of evidence for vitamin D or calcium and BMD was very low.

**Conclusion:**

The impact of micronutrient supplementation on clinical outcomes is unclear, however, monitoring vitamin D levels and calcium intake after SCT should be considered to detect deficiency or inadequate intake.

**Supplementary Information:**

The online version contains supplementary material available at 10.1007/s00520-025-10024-8.

## Introduction

Stem cell transplantation (SCT) has become a common treatment for many haematological malignancies. With the incidence of haematological malignancies increasing, there have been many advances in treatment options [1]. However, recipients of SCT continue to exhibit a significant risk of procedure-related mortality and morbidity, including graft versus host disease (GVHD), infections, and toxicity from conditioning regimens [2]. Adverse transplant-related outcomes occur not only immediately after transplantation but years afterward [3].

Autologous SCT (reinfusion of own cells) and allogeneic SCT (stem cells from a matched donor) have a significant impact on nutrition. Recipients of SCT have an increased risk of micronutrient deficiency and malnutrition due to the conditioning regimens that cause gastrointestinal toxicity such as mucositis, nausea, anorexia and/or associated complications such as GVHD which can inhibit adequate nutrition intake and/or absorption [4–6]. Additionally, the use of restrictive low-bacterial or neutropenic diets can further limit food options. Recent research has focused on challenging historical practices such as neutropenic diets which can negatively impact a patient’s quality of life [7, 8]. Increased clarity on micronutrient supplementation and testing is needed, with some guidelines recommending routine vitamin D and calcium supplements to prevent the loss of bone mineral density (BMD), without guidance on monitoring micronutrient levels [9].

Vitamin D has important immunomodulatory functions that may influence mortality outcomes [10]. A meta-analysis found lower vitamin D status pre SCT was associated with significantly poorer overall survival in autologous and allogeneic SCT patients [10]. The role of micronutrients in the development of GVHD through immune-modulatory effects has been proposed in the literature [11, 12]. A systematic review has evaluated micronutrient supplementation during the first 100 days post-SCT, however, this review excluded patients with GVHD [13]. Several studies have been undertaken to explore the role of vitamin deficiency in the development of acute GVHD [14–16] as well as the effect of vitamin supplementation on clinical outcomes (including acute GVHD) at Day + 100 [17]. However, the prevalence of micronutrient deficiencies in the post-acute phase (> 100 days) and during GVHD at any stage requires further exploration to inform monitoring and supplementation in clinical practice.

This review aimed to synthesise current evidence for vitamin and mineral monitoring and supplementation for patients > 100 days post-SCT or during GVHD, and their associations with any clinical outcomes (such as micronutrient deficiency, BMD, bone fractures, sarcopenia, GVHD, and mortality) to inform clinical practice.

## Methods

This systematic review followed the Preferred Reporting Items for Systematic Reviews and Meta-Analysis (PRISMA) 2020 statement [18]. The review was registered prospectively on PROSPERO on the 23rd of April 2023 (416,776, https://www.crd.york.ac.uk/prospero/display_record.php?RecordID=416776).

### Eligibility Criteria

Studies that met the following criteria were eligible for inclusion: (1) a population including adult patients who underwent their first or second autologous or allogeneic SCT for haematological or non-haematological malignancies; and (2) received micronutrient supplementation and/or monitoring of micronutrient levels more than 100 days post-SCT or during GVHD (present at any stage post-transplant). Studies that examined protein, probiotic, and/or prebiotic supplementation or included paediatric participants was excluded. Randomised control trials, cross-sectional studies, case series, prevalence studies, observational studies, and quasi-experimental studies were all included. Conference abstracts, case studies, letters to the editor, grey literature, and articles where the full text was not available or was not available in English were also excluded.

### Search strategy

Five databases; Embase, Medline, Scopus, Web of Science, and CINAHL, were searched. The initial search was conducted between April 2023 and May 2023 and utilised relevant keywords and subject headings, and was updated in May 2024. Keywords included: antioxidant, nutrient, micronutrient, vitamin, multivitamin, mineral, supplement, calcium, iron, magnesium, phosphate, potassium, sodium, and zinc. The search strategy imposed no restrictions on publication year, study type, geographical location, or setting. The full searches conducted are outlined in Supplementary Materials 1. Reference lists within the included articles were screened for any additional eligible studies.

### Data extraction

Title and abstract screening were conducted independently by two authors utilising the eligibility criteria. Articles that fulfilled the inclusion criteria underwent full-text screening independently by two authors. Disagreement between authors regarding study inclusion was resolved by a third author when required. Data were extracted from the full-text studies by two authors independently and included study location, study design, sample size, sex, age, transplant type, years since SCT, micronutrient(s) of interest, if supplementation was provided, supplementation definition, micronutrient measurement method, measurement time-points, and key outcomes. Disagreements regarding the extracted data were resolved through a discussion between the two authors.

### Data synthesis and interpretation

The Academy of Nutrition and Dietetics checklist was used to assess studies for quality and risk of bias [19]. The Grading of Recommendations, Assessment, Development and Evaluation (GRADE) system [20] was utilised to inform data synthesis and interpretation. Assessments were conducted independently by two authors, and if a consensus could not be reached, a third author was utilised. Where more than one study evaluated the same micronutrient and clinical outcome a narrative synthesis of findings was undertaken. The results were summarised by micronutrient with the studies focusing specifically on patients with GVHD discussed separately where relevant.

## Results

**Fig. 1:**
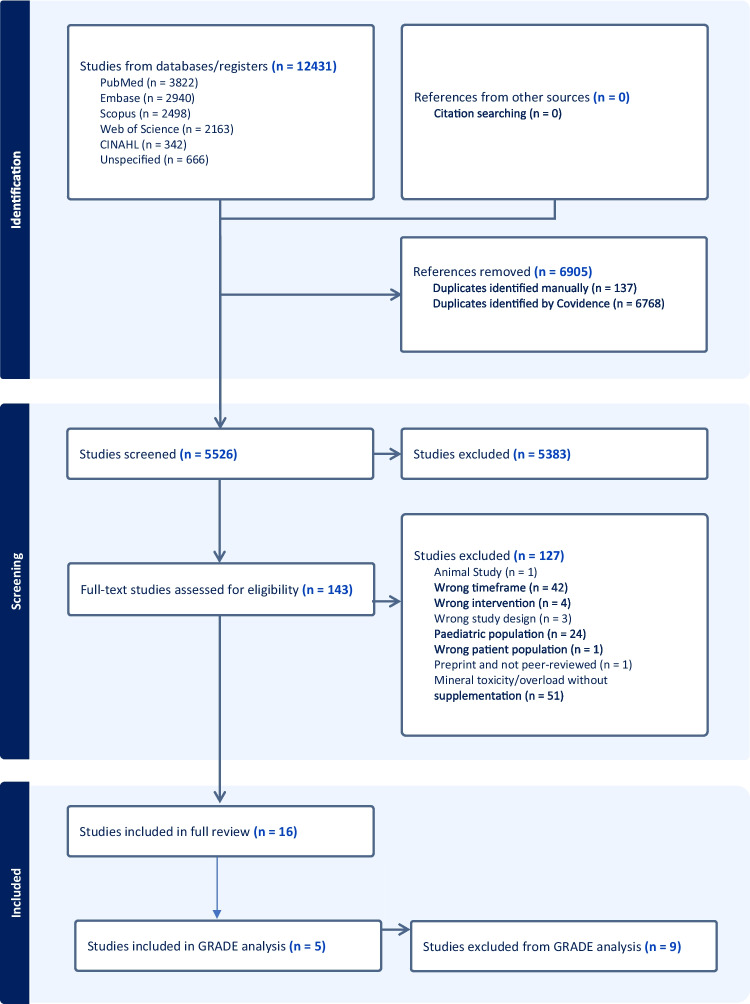
PRISMA Flowchart

​​The search yielded 5526 studies for title and abstract screening after removing 6905 duplicates from the initial 12,431 studies. 143 studies were selected for full-text screening of which 16 studies met the inclusion criteria (Fig. 1). No additional articles were identified when searching the references of the included articles.

A summary of included studies are shown in Table 1. Study sample sizes ranged from nine to 652 participants, with a pooled total of 1,573 participants from all studies [21–36]. Ten of the studies were observational, and six were randomized controlled trials (RCTs). Twelve studies explored micronutrient status or supplementation in patients > 100 days post-SCT [21–26, 28, 29, 31, 32, 34, 36]. Of these, five explored vitamin D [21–23, 28, 29], six vitamin D and calcium [24–26, 31, 32, 34] and one calcium alone [36]. Three studies explored micronutrient status or supplementation during GVHD at any time point after SCT [27, 33, 35]. Of these, one evaluated vitamin D [27], one vitamin D and calcium [33], and one vitamin A [35]. Finally, one study explored vitamin D status in patients > 100 days post-SCT and during GVHD [30]. Four of the six RCT’s investigated the effect of bisphosphonates in combination with vitamin D and calcium supplementation on bone health compared to vitamin D and calcium supplementation alone [24, 25, 31, 34]. The vitamin D and calcium supplementation groups (without bisphosphonates) were included in this review to observe the effect of supplementation on BMD and were classified as a sub-group analysis only [24, 25, 31, 34]. The results of the quality assessment show that all 16 studies received a neutral rating (Supplementary Materials 2) [21–36].

**Table 1:** Studies included in the review of micronutrient monitoring and supplementation >100 days post-SCT and during GVHD: demographics and key findings

Study, study location & study design	Aim of study	Sample size (gender), age, transplant type (%)	Timeframe post-SCT	Presence of GVHD during study period (Y/N)	Micronutrient(s) of interest (what was measured & frequency)	Supplementation observed (Y/N) (type, dose, length)	Vit D deficiency found (Y/N) (prevalence)	Definition of Vit D deficiency	Dietary calcium measured (Y/N)	Outcome measured	Main findings/Other Clinical Outcomes
Baumgartner et al. (2019), Switzerland, Observational (retrospective cohort study) [22]	Evaluate the association of different parameters with the risk of fracture or impaired bone health in recipients of allogeneic transplants	n = 652 (60% male) Age (at transplant): median 49.7 (IQR 39.1–58.8.1.8) Allo: 100%	4.6 (median) IQR 1.7–8.4.7.4 years	(Y) Acute & cGVHD measured. cGVHD present in 346 at 6 years post-SCT (53%)	Vit D (Measurement of Vit D not described, but F/U noted every 3 months post-SCT for the first 2 years & at least every 12 months thereafter)	(Y) Vit D & calcium given to every patient receiving glucocorticoids. Vit D & calcium supplements was regularly implemented during hospital stay & the first year of F/U. (Prevalence, type, dose unknown)	Not described	Not described	(N)	Impaired bone health using BMD. Risk of fractures. (F/U every 3 months post-SCT for the first 2 years & at least every 12 months thereafter)	Vit D deficiency was found to be a significant predictor for impaired bone health during F/U (HR 1.09, 95%CI 1.04–1.15, p = 0.001).Vit D deficiency was found to be a significant predictor for bone fractures during F/U (HR 1.25, 95%CI 1.11–1.41, p < 0.001)
Gubrianska et al. (2019), Sweden, Observational (cross-sectional study) [23]	Evaluate bone status in long-term survivors of allogeneic transplant	n = 20 (60% male) Age: 49 (range 34–69) Allo: 100%	Mean (range): 13.4 (10–15) years	(Y) 18/20 (90%)	Vit D Serum 25(OH)D (nmol/L) (one point in time)	(Y) 15% of patients were taking Vit D supplements at evaluation (type, dose unknown)	(Y) 9/20 (45%)	<50 nmol/L	(N)	BMD (g/cm2) of the femoral neck, total hip, lumbar vertebrae (L1-L4), & total body	No correlation between Vit D levels & BMD Z scores in hip or lumbar spine
Kenny et al. (2019), USA, Non-randomised controlled trial [21]	Generate a process for monitoring & treating Vit D deficiency & determine if therapeutic Vit D levels are attainable post-transplant using a replacement algorithm	n = 144 (55% male) Age: Vit D sufficient 54.7 (11.6); Vit D deficient 56.4 (SD 12.8) Allo: 50% Vit D sufficient, 44% Vit D deficient, Auto: 50% Vit D sufficient, 56% Vit D deficient	Within 6 months	Not described	Vit D Serum 25(OH)D (ng ⁄mL), (measured according to replacement guidelines but within 6 months of SCT)	(Y) phase 2: All patients received Vit D under the ‘Adult Hematopoietic Stem Cell Transplant Vitamin D Replacement Therapy Guideline’ created by authors; supplement level adjusted based on serum Vit D	(Y) 38/144 (26.4%) post supplementation within 6 months of SCT	<20 ng/mL	(N)	None described	Phase 2: 72.9% (n=144) of SCT recipients were Vit D deficient before transplant. Post supplementation the prevalence of Vit D deficiency reduced to 26.4% within 6 months post-SCT. Vit D deficiency in auto patients decreased from 75.6% to 32.1% & in allo patients from 69.7% to 19.7%
Kerschan-Schindl et al. (2004), Austria, Observational (cross-sectional study) [28]	Determine bone metabolism, BMD, & vertebral fractures of long-term survivors of allogeneic transplants	n = 22 (82% male) Age: median 38 (range 22–55) Allo: 100%	Median (range): 6.1 (5.4–17.25.4.25) years	(N)	Vit D Serum 25(OH)D (nmol/L) (one point in time)	(N)	(Y) 3/22 (15%)	<30 nmol/L [Reference range: 30–85 nmol/L]	(N)	BMD (g/cm2) of the lumbar spine (L1-l4), femoral neck, trochanter & intertrochanteric region, ward’s triangle, & total hip	A positive correlation between 25(OH)D & BMD of the lumbar spine was found (r= 0.512, P = 0.036)
Laroche et al. (2012), France, Observational (longitudinal study) [29]	Determine the effect of high-dose chemotherapy & auto SCT on bone turnover & BMD	n = 39 (44% male) Age: median 56 (range 53–61) Auto: 100%	Diagnosis, before, 0.5 & 1 year	Not described	Vit D 25OHD3 (ng ⁄mL) (at diagnosis, before SCT, 6 & 12 months post-SCT)	(N)	(Y) At all timepoints the median Vit D status was found to be deficient (prevalence not described)	<25ng/mL	(N)	BMD (g/cm2) of the lumbar spine, (L1-L4), bilateral total femoral neck, & total body. (At diagnosis, before SCT, 6 & 12 months post- SCT)	Spinal BMD & axial BMD increased significantly from diagnosis to 12 months post-SCT. Femoral neck BMD & appendicular BMD significantly decreased from diagnosis to 6 months post-SCT. Changes in lumbar BMD & total body were not significant over the study period
Kananen et al. (2002), Finland, Observational (cross-sectional study) [26]	Evaluate the BMD & bone turnover of long-term survivors of allogeneic transplant & compare this to the values obtained at 1-year follow-up	n = 27 (48% male) Age: 44 (SD 9) Allo: 100%	Median (range): 6.25 (4.5–8.5) years	Not described	Vit D & calcium Serum 25(OH)D (nmol/L), Serum ionised calcium & dietary calcium (one point in time)	(N)	(Y) 24/27 (89%)	≤37 nmol/L	(Y) Mean (SD): 1100 (600) mg/day	BMD (g/cm2) of the lumbar spine (L1-L4), femoral neck, trochanter, ward’s triangle, & total hip	From 12 months post-SCT to F/U, BMD increased at all sites but was statistically significant at the lumbar spine only (p=0.002)
Pereira et al. (2017), Brazil, Observational (cross-sectional study) [32]	Investigate the prevalence of pre-sarcopenia & BMD in recipients of allogeneic transplants	n = 97 (54% male) Age: mean ± SD (37.4 ± 12.7) Allo: 100%	1–5 years: 50.5% 6–10 years: 15.5% >10 years: 34.0%	(Y) 23/97 (23.7%)	Vit D & calcium Serum 25(OH)D (ng ⁄mL), serum calcium, dietary calcium & vit D (one point in time)	(N)	(Y) 26/97 (37.14%)	<20 ng/mL	(Y) Inadequate in 96.4% patients.	BMD of the lumbar spine, femur (neck & total), & total body. Categorised as 'abnormal' or 'normal' BMD. Sarcopenia	Abnormal BMD was observed in 25% of patients with no statistical significance between patients with adequate vs inadequate intake of both calcium & Vit D. No association between dietary Vit D & BMD. No association between Vit D or calcium (serum or dietary intake) & pre-sarcopenia
Välimäki et al. (1999), Finland, Prospective randomised study [36]	Investigate bone loss following allogeneic BMT & if bone loss can be prevented by calcium with or without calcitonin	n = 44 (47.7% male) Age: mean (S.D) - control: 40 (10), calcium: 40 (8), Ca + calcitonin: 41 (12) Allo: 100%	0–1 year	(Y) Acute GVHD: 21 (47.7%) cGVHD: 22 (50%)	Calcium Serum ionized calcium (before & 3 weeks, 6 weeks, 3 months, 6 months, & 12 months after SCT)	(Y) n= 12 (27%) received calcium [1g twice daily for 12 months] & n=10 (23%) received calcium [1g twice daily for 12 months] plus intranasal calcitonin 400 IU/day for the first month & then 200 IU/day for 11 months	N/A	Not described	(N)	BMD (g/cm2) of the lumbar spine (L1-L4), femoral neck, trochanter, & wards triangle. T scores calculated, but not described in study (before SCT & 6 &12 months)	No statistical significance was found between the groups at all of the measurement sites. No statistical difference for bone loss & bone markers was found between those who took calcium & those who didn't take calcium. Before BMT 39% had osteopenia & osteoporosis at the lumbar spine & this increased to 47% at the end of the study. Before BMT 25% had osteopenia & osteoporosis at the femoral neck & this increased to 58% at the end of the study
Hari et al. (2013), Canada & USA,Phase II, Randomised, open-label trial [24]	Complete a trial of intravenous zoledronic acid & evaluate the effect on BMD loss in adult recipients of allogeneic transplants	Subgroup analysis (Did not receive IV zoledronic acid; received calcium & Vit D only): n = 29 (25% male) Age: median 51 (range 28–70) Allo: 100%	0–1 year	(Y) Acute GVHD: 6 (21%). cGVHD: 8 (28%)	Vit D & calcium Serum calcium (baseline & at 12 months post-SCT)	(Y) All participants received 1000 mg of calcium carbonate + 400–500 IU of Vit D daily	Not described	Not described	(N)	Change in BMD (g/cm2). T & Z scores calculated. Fractures. Mortality. (baseline & at 12 months post-SCT)	BMD of the lumbar spine & femoral neck found that both decreased from baseline to 12 months post-SCT at a mean of −0.057 (range −0.215 to 0.093) & −0.054 (−0.36 to 0.073) respectively
Kananen et al. (2005), Finland, Randomised controlled trial [25]	Determine if additional IV pamidronate would prevent bone loss in recipients of SCT more effectively than calcium, vitamin D, & sex steroid replacement therapy alone	Subgroup analysis (Did not receive IV pamidronate; received calcium, Vit D, & HRT only): n = 37 (49% male) Age: 44 (10) (mean (SD))Allo: 100%	0–1 year	(Y) Acute GVHD: 9 (24.3%). cGVHD: 14 (37.8%)	Vit D & calcium: Serum ionized calcium (before & 1, 3, 6, & 12 months after SCT). Vit D levels not reported	(Y) All participants received 1000 mg of calcium carbonate + 800 IU of Vit D daily	Not described	Not described	Calcium intake (mg/day) from dairy products at baseline: (920 [mean] (630 [SD])	Change in BMD (g/cm2) of the lumbar spine (lumbar vertebrae L1–L4) & femoral sites (femoral neck, trochanter, & total hip)(before & 6 & 12 months post-SCT)	After 12 months, BMD decreased at the: lumbar spine by 2.9% (P=0.031), femoral neck by 6.2% (P < 0.001),trochanter by 9.8% (P <0.001), & total hip by 7.8% (P< 0.001)
Lu et al. (2016), USA, Randomised controlled trial [31]	Determine the effects of ibandronate on bone loss following allogeneic transplantation	Subgroup analysis (Did not receive IV ibandronate; received calcium & Vit D only): n = 39 (72% male) Age: 50.2 (12.9) (mean (SD))Allo: 100%	0–1 year	Not described	Vit D & calcium Serum 25(OH)D (ng ⁄mL) (baseline & 12 month after SCT) & serum calcium (3 & 12 months after SCT)	(Y) All patients received 500 mg of calcium + 800 IU of Vit D daily for 12 months. As part of the pre-transplant workup (before study) all patients with Vit D levels >20ng/ml were given 50,000 IU for 12 weeks	50 patients (64%) were deficient in Vit D at study baseline (<100 days)	<20 ng/mL	(N)	Change in BMD (g/cm2) of the lumbar spine (L1-L4), femoral neck & total hip (6 & 12 months post-SCT)	6 months: significant BMD loss [mean (SD)] at lumbar spine −2.61 (4.2) [p=0.004], femoral neck −4.85 (5.37) [p<0.001], & total hip −4.72 (4.32) [p<0.001]. 12 months: significant BMD loss [mean (SD)] in femoral neck-5.20 (5.25) [p=0.001] & total hip −5.68 (5.15) [p<0.001]
Tauchmanovà et al. (2003), Italy, Prospective randomised study [34]	Determine the effect of risedronate on bone mass turnover in allogeneic transplant recipients	Subgroup analysis (Did not receive oral risedronate; received calcium & Vit D only): n = 17 (47% male) Age: 33.2 (10.4) (mean (SD))Allo: 100%	0–1 year	Not described	Vit D & calcium Serum calcium (mmol/L), urinary calcium (measured at study entry, & after 3,6, & 12 months)	(Y) All patients in the subgroup received 1 g calcium & 800 IU Vit D daily for 6 months over study period	Not described	Not described	(N)	BMD g/cm2) of the lumbar spine (L1-L4), femoral neck, & total hip. (At baseline, & after 6 & 12/13 months)	At 6 months: lumbar BMD decreased by 4.3±1.5% (P<0.05), femoral neck BMD decreased by 4.3±2.1%; P<0.05); total hip BMD decrease by 4.1±1.6% (P<0.05). From 6–12 months: lumbar BMD increased by 1.1±1.4%, (−3.1±1.4% of baseline); femoral, neck & total hip BMD did not change significantly
Katić et al. (2016), Croatia, Observational (cross-sectional with a longitudinal survival follow-up) [27]	Determining factors associated with Vit D status in patients with chronic GVHD & investigate the association between Vit D, cGVHD characteristics & clinical outcomes.	n = 310 (55% male) Age: median 48 (IQR 36–57) Allo: 100%	Median (IQR): 3.0 (1.9–5.9) years	(Y) 100%	Vit D Serum 25(OH)D (ng/mL) (one point in time)	(Y) 56% (n=175) of patients took a Vit D or multivitamin supplement or both 1 month prior or currently at evaluation (type, dose unknown)	(Y) 69/310 (22.3%)	≤20ng/mL	(N)	Major cGVHD outcomes (at one point in time) Mortality (longitudinal F/U)	No association between Vit D level & major cGVHD outcomes. No significant association was found between patients with Vit D ≤20 ng/mL & decreased survival (p=0.042; significance level set at p=0.005)
Stern et al. (1996), USA, Observational (longitudinal pilot study) [33]	Determine whether allogeneic transplant recipients receiving treatment for GVHD experienced loss of BMD & if dietary intake/biochemical parameters contributed to the development of osteoporosis	n = 9 (67% male) Age: 39 (range 31–47) Type of transplant not specified	~0.25-1year	cGVHD: 100%	Vit D & calcium 25(OH)D & (1,25(OH)₂D) (before & 3 & 12 months post-SCT)	(Y) Multiple vitamin supplement given to all patients containing 400 IU Vit D. 1 patient (11%) received Vit D through TPN (dose unknown)	25(OH)D: normal levels throughout study period. 1,25(OH)₂D: mean level below normal range for males & low normal for females at enrolment. Low normal at 12 months for males & females	Normal 25(OH)D2 range: 17–50 ng/mL	Dietary calcium intake ≥ 1200 mg in at least 3/5 evaluations	BMD (g/cm2) of the lumbar spine (L1-4) & regional bone mass of the wrist. (At study entry: 3 months post-SCT & F/U: 12 months post-SCT)	No association between Vit D2, BMD changes, & urinary calcium excretion. 6/9 (67%) of patients had significant bone loss in the lumbar spine
Tong et al. (2017), China, Observational (cohort study) [35]	Evaluate the association between serum Vit A & ocular manifestations of GVHD	GVHD subgroup: n = 19 (55% male of entire cohort)Age: 32.79 ±10.40 Allo: 100%	cGVHD onset 4–17 months post-SCT	(Y) 100%	Serum Vit A (before SCT, 3 months after SCT & during GVHD)	(N) Vit A ointment used when deficiency present (prevalence, type, dose unknown)	N/A	Not described	(N)	Grade of ocular GVHD (before SCT, 3 months after SCT, & during cGVHD)	Grade of ocular GVHD higher in patients with Vit A deficiency (r = 0.830, P = 0.000). Prevalence of Vit A deficiency not described. Serum Vit A slightly increased when GVHD occurred by 0.965 ± 0.304 μg/ml (t = 2.553; P = 0.020). When liver GVHD occurred serum vit A levels decreased to 0.18 +/- 0.03 (t=6.003; P =0.004)
Ljubas Kelecic et al. (2020), Croatia, Observational (cross-sectional study) [30]	Evaluate the frequency & characteristics of sarcopenia among recipients of allogeneic transplantation focusing on chronic graft-versus-host disease	n = 73 (51% male) Age: median 47 (range 20–66) Allo: 100%	Medium (IQR): 1.3 (0.5 - 2.7) years (>100 days). Median (IQR): 1.4 (0.5–4.5) years	(Y) 45/73 (61.6%)	Vit D Serum 25(OH)D (nmol/L) (one point in time)	(N)	(Y) 40/73 (54.8%) of entire cohort. 12/28 (42.9%) patients >100 days & 28/45 (62.2%) in cGVHD patients	<50 nmol/L	(N)	Sarcopenia: Body composition & hand grip strength (one point in time)	No statistically significant difference between sarcopenia & patients with or without GVHD. No significant correlation between serum Vit D & sarcopenia. A positive association between Vit D levels & reduced physical function (2MWT) was found in patients with cGVHD. Malnutrition was found more often in allo-SCT patients with sarcopenia (P = 0.004). Malnutrition prevalence: 42.5% (n=31). Patients with cGVHD were significantly more likely to be malnourished than patients without cGVHD

BMT = Bone marrow transplant; Allo = Allogeneic Transplant; Auto = Autologous Transplant; SCT = Stem cell transplant; GVHD = Graft-versus-host-disease; cGVHD = Chronic Graft-versus-host-disease; BMD = Bone Mineral Density; Vit D = Vitamin D; Vit A = Vitamin A; F/U = Follow-up; TPN = Total Parenteral Nutrition; HRT = Hormone Replacement Therapy, 2MWT= 2-minute walk test

### Vitamin D

Vitamin D was the focus of 14 of the 16 studies [21–34]. Nine studies provided or observed existing vitamin D supplementation [21–25, 27, 31, 33, 34], seven described the prevalence of vitamin D deficiency [21, 23, 26–28, 30, 32], one reported deficiency rates < 100 days post-SCT [31], and two studies reported only mean or median vitamin D levels [29, 33]. Four studies reported on supplementation in conjunction with vitamin D deficiency rates or the mean vitamin D levels of the patient group [21, 23, 27, 33].

### Vitamin D deficiency and supplementation in patients > 100 days post-SCT 

In patients > 100 days post-SCT, five observational studies [23, 26, 28, 30, 32] and one non-randomised trial [21] reported the prevalence of vitamin D deficiency to range from 15 to 89%. The timeframe for testing for deficiency post-SCT was variable, ranging from within the first six months [21] to over six months [30] and greater than one year [23, 26, 28, 32]. Two of the six studies describing the prevalence of vitamin D deficiency also detailed that supplementation was observed or provided [21, 23]. An observational study reported 15% of patients were taking a vitamin D supplement at the time of evaluation with a reported deficiency rate of 45% [23]. In the second study, 72.9% of participants were vitamin D deficient before SCT, and after supplementation, deficiency reduced to 26% within six months of SCT [21].

### Vitamin D deficiency and supplementation in patients during GVHD

Three observational studies explored vitamin D levels in patients with GVHD [27, 30, 33]. Two studies reported on the prevalence of vitamin D deficiency in patients with GVHD between six months to five years post-SCT [27, 30]. One study reported a deficiency rate of 62.2% [30]. The second found the prevalence of vitamin D deficiency to be 22.3%, however, 42.9% of patients received vitamin D and/or a multivitamin within the previous month [27]. The third study did not report the prevalence of vitamin D deficiency, however, mean vitamin D was within normal parameters at enrolment (three month-post-SCT) and follow-up (one-year post-SCT; value not reported) [33]. Mean vitamin D2 levels were below range at enrolment but increased to normal range one-year post-SCT for males, while females maintained vitamin D2 levels within normal range throughout the study (normal range 17–50 ng/ml) [33]. Participants were provided a multivitamin supplement containing 400 IU (10mcg) of vitamin D and one patient received vitamin D via parenteral nutrition (PN), however, the frequency and length of supplementation were unclear [33].

### Vitamin D, BMD and fractures in patients > 100 days post-SCT

Four observational studies reported variable results regarding the association between vitamin D levels and BMD in patients > 100 days post-SCT [22, 23, 28, 32]. Two studies evaluating patients up to 13 years post-SCT found no association between BMD and vitamin D status or dietary vitamin D [23, 32]. Conversely, Kerschan-Schindl et al. [28] found a positive correlation between vitamin D and BMD of the lumbar spine, and Baumgartner et al. [22] found that vitamin D deficiency during follow-up was significantly associated with impaired bone health.

One large observational study by Baumgartner et al. [22] found vitamin D deficiency was significantly associated with an increased risk of fracture at a median 4.6 years post-SCT, with 53% of patients at six years post-SCT diagnosed with chronic GVHD. Acute and chronic GVHD was significantly associated with an increased risk of fracture (P = < 0.001; P = 0.028 respectively) in conjunction with the diagnosis of osteoporosis and osteopenia at follow-up (P = 0.001; P = 0.004 respectively) [22].​ Another study evaluated the incidence of fractures and BMD and vitamin D levels [26]. Despite 89% of patients having a vitamin D deficiency and no known supplementation, BMD improved at all sites from 12 months to a median of 6.25 years post-SCT but this was statistically significant at the lumbar spine only [26]. In the study by Laroche et al. [29] while at all time-points the median vitamin D status was found to be deficient and vitamin D supplements were not taken, BMD significantly increased at the lumbar spine and axial skeleton 12 months post-SCT.

### Vitamin D and BMD in patients during GVHD

One pilot study explored the association between vitamin D and BMD in patients with GVHD up to one-year post-SCT [33]. At 12 months post-SCT, patients had a mean low normal vitamin D3 (1,25 (OH)_2_D (22.1 ± 2.4 (normal range 17–50 ng/ml)) and mean normal vitamin D (25(OH)D) (value not reported) [33]. BMD of the lumbar spine significantly decreased in 75% of patients from three to 12 months post-SCT​, and 12.5% of patients had significant bone loss at the wrist. While it was reported that there did not appear to be an association between vitamin D and BMD, statistical analysis was not reported [33].

### Vitamin D and other clinical outcomes

​​Two observational studies reported on vitamin D and sarcopenia for patients > 100 days or during GVHD [30, 32]. No association between pre-sarcopenia or sarcopenia and vitamin D levels ≥ six months post-SCT was found [30, 32]. However, in the study by Ljubas Kelecic et al. [30] a negative association between the 2-min walking test (2MWT) and vitamin D levels was found in patients with chronic GVHD (rho −0.326, p = 0.0489). The study by Katić et al. [27] found no association between vitamin D and mortality in patients with GVHD at a median of three years post-SCT.

### Calcium

Calcium was a micronutrient of interest for eight of the 16 studies [24–26, 31–34, 36]. Three studies were observational [26, 32, 33], one was an RCT [36], and four were included in the sub-group analysis [24, 25, 31, 34].

### Calcium intake and supplementation in patients >100 days or during GVHD

Two studies evaluated dietary calcium intake, one in patients >100 days post-SCT [32] and one in patients with GVHD [33]. In patients >100 days post-SCT, mean daily intake of calcium was 456 ± 347 mg in patients evaluated one to over 10 years, where 95% of patients consumed an inadequate intake of calcium, and 23.7% had GVHD [32]. In contrast, oral calcium intake was found to be ≥ 1200 mg/day in patients with GVHD at three of the five follow-up evaluations within the first year post-SCT [33]. Calcium supplementation was not described by Pereira et al. [32], and while a multivitamin supplement was provided in the study by Stern et al. [33], calcium dose was not reported.

### Calcium and BMD in patients > 100 days post-SCT or during GVHD

One observational ​study and one ​RCT investigated calcium intake and BMD in patients > 100 days post-SCT [32, 36]. Pereira et al. [32] found no statistically significant association between mean calcium intake and BMD from one to > 10 years post-SCT. The RCT evaluated patients in the first year post-SCT and randomly assigned patients to a no-treatment group, oral calcium group, and calcium plus calcitonin ​group [36]. The prevalence of osteopenia and osteoporosis for all patients one-year post-SCT increased by 11% and 33% at the lumbar and femoral neck and 40.9—54.5% of patients developed GVHD [36]. No statistically significant BMD changes amongst groups were found, or between the two calcium supplementation groups and the group that took no supplement [36].

Stern et al. [33] assessed calcium and BMD during GVHD. 75% of participants had a significant loss of BMD in the lumbar spine, and 12.5% had loss in the wrist, however no statistical analysis was reported on associations between calcium measurements and BMD [33].

### Calcium and Vitamin D supplementation in sub-group analysis

Four studies provided calcium and vitamin D supplementation and measured BMD [24, 25, 31, 34]. All studies evaluated serum or ionised calcium, and one study [31]assessed vitamin D at baseline only (< 100 days). Three of the four studies evaluated patients from baseline to 12 months post-SCT and found the mean or median BMD values decreased at all sites despite supplementation [24, 25, 31]. Tauchmanovà et al. [34] evaluated patients 1.5 years post-SCT and found all BMD sites decreased at follow-up but were significant at the total hip and femoral neck only.

### GRADE certainty of evidence

Five studies that evaluated patients > 100 days post-SCT were included in the GRADE assessment (Table 2) [22, 23, 28, 32, 36]. The remaining studies were not included in the GRADE assessment as clinical outcomes were either explored by only one study or not evaluated. The GRADE certainty of evidence for BMD and vitamin D was rated very low, with studies downgraded due to a very serious risk of inconsistency and indirectness, and serious risk of bias and imprecision (Table 2). The GRADE certainty of evidence between calcium and BMD was rated very low due to the very serious risk of indirectness, imprecision, and serious risk of bias (Table 2).

**Table 2:** GRADE_a_ certainty of patient outcomes in five included studies of the vitamin and mineral requirements in patients > 100 days post-SCT and during GVHD

	Study Design	Certainty Assessment					
		Risk of Bias	Inconsistency	Indirectness	Imprecision	Publication Bias	Certainty
Vitamin D	Observational	Serious	Very Serious	Very Serious	Serious	Not suspected	Very Low
BMD [22, 23, 28, 32]							
Calcium	Observational, RCT	Serious	Not suspected	Very Serious	Very Serious	Not suspected	Very Low
BMD [32, 36]							
^a^GRADE = Grading of Recommendations Assessment, Development, and Evaluation							

## Discussion

This review aimed to synthesise current evidence for vitamin and mineral monitoring and supplementation for patients > 100 days post-SCT or during GVHD, and their associations with any clinical outcomes to inform clinical practice. To our knowledge, this is the first systematic review to evaluate the micronutrient requirements for SCT patients in the post-acute transplant phase. Overall, the review found limited data and an unclear association between micronutrient deficiency or micronutrient supplementation and the clinical outcomes of interest (BMD, fractures, sarcopenia, major chronic GVHD outcomes, and mortality). Furthermore, the GRADE certainty of evidence assessment for vitamin D or calcium and BMD was rated as very low. BMD was the most commonly reported clinical outcome, and calcium and vitamin D were the most frequently studied micronutrients. Vitamin D deficiency was reported to be between 15—89% for patients > 100 days post-SCT, and 22.3%—62.2% for patients during GVHD.

### Vitamin D deficiency

The prevalence of vitamin D deficiency in patients > 100 days post-SCT or during GVHD varied across studies. Variability may in part be due to vitamin D supplementation practices and variable definitions of vitamin D deficiency. Segon et al. [13] evaluated patients’ vitamin D status in the acute post-SCT phase (< 100 days post-SCT) and found that the definition and prevalence of vitamin D deficiency varied at 23.5%, (< 10 ng/ml), 60% (< 25 ng/mL), and 56% (< 52 nmol/L). Inconsistency in findings may also be attributable to the type, dose, and frequency of vitamin D prescribed and patient adherence. Kenny et al. [21] strictly adhered to guidelines, while in other studies the actual doses consumed were unclear [23, 27]. Seasonality may also have contributed to variability of vitamin D deficiency across studies.

### Vitamin D and Calcium Supplementation

Recommendations for the general population with risk factors for vitamin D deficiency are 1000–2000 IU (25-50mcg) of vitamin D3 daily, with many other countries recommending between 400–2000 IU (10-50mcg) daily [37, 38]. Moderate to severe vitamin D deficiency recommendations are 3000–4000 IU (75-100mcg) of vitamin D3 for 6–12 weeks, followed by 1000–2000 IU (25-50mcg) daily thereafter [37]. Patients post-SCT have increased risk factors for vitamin D deficiency due to increased time spent indoors following increased photosensitivity, steroid usage, underlying disease, toxicity, GVHD, and subsequent organ failure. Therefore, more aggressive vitamin D supplementation may be theoretically indicated in this population. However, variability in vitamin D deficiency rates within each study supports individualised monitoring of levels and supplementation. Furthermore, inadequate dietary intake of calcium may be experienced by this patient group due to malnutrition, and/or patient preference for low calcium-containing foods [39]. Calcium intake should be estimated via a food frequency survey or thorough diet history by a qualified Dietitian to determine if calcium supplementation is also required post-SCT. Thorough assessment of medications, specifically proton pump inhibitors (PPI), which may impact calcium absorption through gastric acid suppression [40], should be considered when choosing an appropriate calcium supplement.

In the study by Ros-soto et al. [41] 326 allogeneic transplant centres in 42 countries were surveyed to evaluate monitoring, prescription, and follow-up practices for vitamin D deficiency and supplementation. The median loading dose for patients found to be vitamin D deficient was 2000 IU (50mcg) (range 7-500mcg; 286–20,000 IU), for a median of six weeks (range 1–52) [41]. Following this, a median maintenance dose of 800 IU (20mcg) (range 67–10,000; 1.6-250mcg) was prescribed and discontinued when adequate vitamin D levels were achieved or DEXA returned to normal or symptomatic improvements were reported, or steroids were ceased [41]. Vitamin D Replacement Therapy Guidelines recommend D2 50,000 IU (1250mcg) once weekly for six weeks when vitamin D deficient (< 20 ng/mL), and D2 50,000 IU (1250mcg) once fortnightly or D3 2000 IU (50mcg) daily when levels are 20–29 ng/ml [21]. High-dose supplementation of 5000 IU (125mcg) daily has been associated with reduced prevalence of GVHD [42]. It was also found that 52% of centres prescribed calcium supplementation in conjunction with vitamin D [41].

The frequency of vitamin D status monitoring varied from three-monthly (39%), six-monthly (24%), and yearly (18%), and additional monitoring during GVHD was not described [41]. Hong et al. [42] recommend checking vitamin D levels every 3 months, and rechecking if levels were tested greater than six weeks ago for patients with GVHD. The Vitamin D Replacement Therapy Guidelines recommends that levels should be checked three-monthly or at least annually when vitamin D levels are sufficient [21]. Katić et al. [27] found that nutritional status and vitamin D status were positively associated, and thus vitamin D testing should also be performed in malnourished patients. While life-long monitoring is prevalent in some centres (57%) [41] it has been suggested that when vitamin D status is found to be > 30 ng/ml at two consecutive assessments and the patient has ceased steroids, vitamin D deficiency monitoring can be discontinued [42] Additionally, it has been found that the greater the time post-SCT, the lesser the prevalence and severity of vitamin D deficiency, however, older age also becomes a risk factor for vitamin D deficiency [42].

### Vitamin D, calcium, and BMD

Conflicting associations between vitamin D levels or supplementation and BMD were found, and no association was found between calcium intake or supplementation and BMD, which may be due to the type of transplantation received. The study by Laroche et al. [29] was the only study that evaluated patients undergoing autologous SCT and reported BMD increased over 12 months. Due to the lack of GVHD post autologous transplant, patients are not susceptible to organ and tissue damage and harsh steroid therapies that are associated with GVHD. In the study by Lee et al. [43] a significant positive correlation was found between steroid dose and bone loss in the lumbar spine. Studies included in this review found that GVHD was prevalent in patients > 100 days post-SCT and therefore steroid medication may also impact BMD [22–25, 30, 32, 36].

Other potential factors impacting skeletal recovery are the effect of myeloablative therapy, GVHD, immunosuppressants, and corticosteroids on sexual hormones [44–47]. Early menopause and gonadal failure result in the reduction of sexual hormones and can impact bone health if not replaced [44–47]. In a retrospective study, female SCT patients who received hormone replacement therapy showed significant increases in BMD despite the presence of GVHD and steroid usage two years post-SCT [48]. Furthermore, RCTs have shown that bisphosphonates may improve BMD outcomes [24, 25, 31, 34].

Finally, it is well-known that weight-bearing exercise demonstrates the preservation and recovery of BMD [49]. A study found that patients who had undergone SCT as a child who underwent a 12-week intensive exercise regime showed an increase in bone formation markers, but no changes in BMD [50]. However, physical activity was not assessed in the majority of studies included in this review and therefore it is unknown whether a similar impact would be observed in adults post-SCT [51].

### Limitations

This review was limited by the quality of the available evidence as studies were rated neutral with a moderate risk of bias and a very low certainty of evidence with several studies not eligible for GRADE analysis. Study heterogeneity affected data interpretation and contributed to inconsistent results. Variability arose from differences in time-point assessments post-SCT, malignant versus non-malignant diagnoses, and myeloablative versus non-myeloablative conditioning regimens, among other factors. Furthermore, some studies lacked complete statistical analyses and information about the type, dose, and/or frequency of supplementation. Studies that evaluated BMD varied in site measurements and if calculations of Z and T scores were performed[22–26, 28, 29, 31–34, 36]. Few studies evaluated dietary calcium intake with some only reporting serum calcium which is not an indicator of bone health [39]. Studies that evaluated patients > 100 days post-SCT were shown to contain a high prevalence of patients with GVHD which may impact results and studies that only included participants with GVHD were scarce. Gastrointestinal GVHD in particular may have impacted on micronutrient absorption. Overall, the paucity of literature limited subgroup analysis. Lastly, the search resulted in predominantly vitamin D studies which indicated a lack of evidence for other micronutrients. Further research is required to explore other micronutrients such as Vitamin A, for which only one study was identified [35]. The emerging literature on other fat-soluble vitamins also needs consideration, and this review is applicable to adult populations only [52].

## Conclusion

The impact of micronutrient supplementation on clinical outcomes is unclear with GRADE certainty of evidence for vitamin D or calcium and BMD rated as very low. While the monitoring of vitamin D levels and calcium intake after transplantation should be considered to detect deficiency or inadequate intake, the most appropriate timeframe for retesting is unclear. In situations such as bone mineral density concerns, limited sun exposure, malabsorption or long-term steroid medication use, more frequent testing may be indicated. The definition of vitamin D deficiency is not consistent and thus consensus for the definition will assist with drawing more reliable and valid conclusions. Further high-quality studies evaluating the prevalence of deficiency for other micronutrients and supplementation outcomes in patients > 100 days post-SCT and during GVHD are required to examine the impact on post-transplant outcomes.

## Supplementary Information

Below is the link to the electronic supplementary material.

ESM 1 (28.5 KB DOCX)

## Data Availability

The authors declare they have a file with all data utilised to conduct this review. Included peer-reviewed articles can be accessed via relevant publishers.
