# Supplementary material for: Micronutrient requirements for stem cell transplantation patients > 100 days after transplant and during graft versus host disease: a systematic review
Source: Support Care Cancer. 2025 Nov 7;33(12):1025. doi: 10.1007/s00520-025-10024-8 (PMC12594721; doi:10.1007/s00520-025-10024-8)
Supplement: Supplementary file 1 — (28.5 KB DOCX) [file 520_2025_10024_MOESM1_ESM.docx]

**Supplementary Materials**

1. **Embase Search Strategy**

| #1 | 'trace element'/mj OR 'vitamin'/mj OR 'mineral'/mj OR 'vitamin deficiency'/mj OR 'mineral deficiency'/mj OR 'micronutrient intake'/mj OR 'antioxidant'/mj OR 'vitamin supplementation'/mj OR 'mineral supplementation'/mj OR antioxidant*:ti,ab OR avitaminosis:ti,ab OR 'microelement':ti,ab OR 'nutrient*':ti,ab OR 'micronutrient*':ti,ab OR 'micro nutrient*':ti,ab OR 'vitamin*':ti,ab OR 'multivitamin*':ti,ab OR 'provitamin*':ti,ab OR 'mineral*':ti,ab OR 'trace element*':ti,ab OR 'supplement*':ti,ab OR 'deficienc*':ti,ab OR 'nutritional deficienc*':ti,ab OR 'carotenoid*':ti,ab OR 'beta carotene':ti,ab OR 'alpha carotene':ti,ab OR 'retinol':ti,ab OR 'retinal*':ti,ab OR 'retinyl ester*':ti,ab OR 'retinoic':ti,ab OR 'thiamin*':ti,ab OR 'riboflavin':ti,ab OR 'niacin':ti,ab OR 'folate':ti,ab OR 'folic':ti,ab OR 'pantothenic acid':ti,ab OR 'biotin':ti,ab OR 'choline':ti,ab OR 'ascorbic acid':ti,ab OR 'calciferol':ti,ab OR 'cholecalciferol':ti,ab OR 'ergocalciferol':ti,ab OR 'tocopherol':ti,ab OR 'alpha-tocopherol':ti,ab OR 'phytonadione':ti,ab OR 'phytomenadione':ti,ab OR 'phylloquinone':ti,ab OR 'menaquinone':ti,ab OR 'menadione':ti,ab OR 'calcium':ti,ab OR 'chromium':ti,ab OR 'copper':ti,ab OR 'fluoride':ti,ab OR 'iodine':ti,ab OR 'iodide':ti,ab OR 'iron':ti,ab OR 'ferrous':ti,ab OR 'ferric':ti,ab OR 'magnesium':ti,ab OR 'manganese':ti,ab OR 'molybdenum':ti,ab OR 'phosphorus':ti,ab OR 'phosphate*':ti,ab OR 'phosphoric':ti,ab OR 'potassium':ti,ab OR 'selenium':ti,ab OR 'selenomethionine':ti,ab OR 'sodium':ti,ab OR 'zinc':ti,ab OR 'cobalamin':ti,ab OR 'hydroxycobalamin':ti,ab OR 'cyanocobalamin':ti,ab OR 'pyridoxine':ti,ab OR 'pyridoxal':ti,ab OR 'pyridoxamine':ti,ab |
| --- | --- |
| #2 | 'stem cell transplantation'/mj OR 'bone marrow transplantation'/mj OR 'graft versus host reaction'/mj OR ‘allogeneic stem cell transplantation’/mj OR ‘autologous stem cell transplantation’/mj OR 'stem cell transplant*':ti OR 'stem cell graft*':ti OR 'bone marrow transplant*':ti OR 'bone marrow cell transplant*':ti OR 'bone marrow graft*':ti OR 'hematopoietic transplant*':ti OR 'haematopoietic transplant*':ti OR 'hematopoietic cell transplant*':ti OR 'haematopoietic cell transplant*':ti OR 'graft versus host reaction':ti OR 'graft vs host reaction':ti OR 'graft v host reaction':ti OR 'graft versus host disease':ti OR 'graft vs host disease':ti OR 'graft v host disease':ti OR 'gvhd':ti |
| #3 | #1 AND #2 |
| #4 | #3 NOT ('animal experiment'/de NOT ('human experiment'/de OR 'human'/de)) |
| #5 | #4 AND [english]/lim AND ([article]/lim OR [article in press]/lim) |

1. **CINAHL Search Strategy**

| #1 | (MM "Vitamins") OR (MM "Minerals") OR (MM "Trace Elements") OR (MM "Avitaminosis") OR (MM "Micronutrients") OR (MM "Antioxidants") OR (MM "Dietary Supplementation")  OR “antioxidant*” OR avitaminosis OR microelement OR “nutrient*” OR “micronutrient*” OR “micro nutrient*” OR “vitamin*” OR “multivitamin*” OR “provitamin*” OR “mineral*” OR “trace element*” OR “supplement*” OR “deficienc*”:ti,ab OR “nutritional deficienc*”:ti,ab OR “carotenoid*” OR “beta carotene” OR “alpha carotene” OR retinol OR “retinal*” OR “retinyl ester*” OR “retinoic” OR “thiamin*” OR Riboflavin OR Niacin OR Folate OR Folic OR “Pantothenic acid” OR Biotin OR Choline OR “ascorbic acid” OR calciferol OR cholecalciferol OR ergocalciferol OR tocopherol OR “alpha-tocopherol” OR phytonadione OR phytomenadione OR phylloquinone OR menaquinone OR menadione OR Calcium OR Chromium OR Copper OR Fluoride OR Iodine OR Iodide OR Iron OR Ferrous OR Ferric OR Magnesium OR Manganese OR Molybdenum OR Phosphorus OR “Phosphate*” OR Phosphoric OR Potassium OR Selenium OR Selenomethionine OR Sodium OR Zinc OR cobalamin OR hydroxycobalamin OR cyanocobalamin OR Pyridoxine OR Pyridoxal OR Pyridoxamine (TI)  OR “antioxidant*” OR avitaminosis OR microelement OR “nutrient*” OR “micronutrient*” OR “micro nutrient*” OR “vitamin*” OR “multivitamin*” OR “provitamin*” OR “mineral*” OR “trace element*” OR “supplement*” OR “deficienc*”:ti,ab OR “nutritional deficienc*”:ti,ab OR “carotenoid*” OR “beta carotene” OR “alpha carotene” OR retinol OR “retinal*” OR “retinyl ester*” OR “retinoic” OR “thiamin*” OR Riboflavin OR Niacin OR Folate OR Folic OR “Pantothenic acid” OR Biotin OR Choline OR “ascorbic acid” OR calciferol OR cholecalciferol OR ergocalciferol OR tocopherol OR “alpha-tocopherol” OR phytonadione OR phytomenadione OR phylloquinone OR menaquinone OR menadione OR Calcium OR Chromium OR Copper OR Fluoride OR Iodine OR Iodide OR Iron OR Ferrous OR Ferric OR Magnesium OR Manganese OR Molybdenum OR Phosphorus OR “Phosphate*” OR Phosphoric OR Potassium OR Selenium OR Selenomethionine OR Sodium OR Zinc OR cobalamin OR hydroxycobalamin OR cyanocobalamin OR Pyridoxine OR Pyridoxal OR Pyridoxamine (AB) |
| --- | --- |
| #2 | (MM "Hematopoietic Stem Cell Transplantation") OR (MM "Bone Marrow Transplantation") OR (MM "Bone Marrow Transplantation, Allogeneic") OR (MM "Bone Marrow Transplantation, Autologous") OR (MM "Graft Versus Host Disease") OR (MM "Transfusion-Associated Graft Versus Host Disease") OR (MM "Host Versus Graft Reaction")  OR “stem cell transplant*” OR “stem cell graft*” OR “bone marrow transplant*” OR “bone marrow cell transplant*” OR “bone marrow graft*” OR “hematopoietic transplant*” OR “haematopoietic transplant*” OR “hematopoietic cell transplant*” OR “haematopoietic cell transplant*” OR “graft versus host reaction” OR “graft vs host reaction” OR “graft v host reaction” OR “graft versus host disease” OR “graft vs host disease” OR “graft v host disease” OR GVHD (TI) |
| #3 | #1 AND #2 |
| #5 | #3 NOT (((MH “Animals+”) OR (MH “Animal Studies”) OR (TI ‘animal model*”)) NOT (MH “human”)) |
| #6 | Filters applied: English |

1. **Scopus Search Strategy**

| #1 | TITLE-ABS ( antioxidant* OR avitaminosis OR microelement OR "nutrient*" OR "micronutrient*" OR "micro nutrient*" OR "vitamin*" OR "multivitamin*" OR "provitamin*" OR "mineral*" OR "trace element*" OR "supplement*" OR "deficienc*" OR “nutritional deficienc*” OR "carotenoid*" OR "beta carotene" OR "alpha carotene" OR retinol OR "retinal*" OR "retinyl ester*" OR "retinoic" OR thiamin* OR riboflavin OR niacin OR folate OR folic OR "pantothenic acid" OR biotin OR choline OR "ascorbic acid" OR calciferol OR cholecalciferol OR ergocalciferol OR tocopherol OR "alpha tocopherol" OR phytonadione OR phytomenadione OR phylloquinone OR menaquinone OR menadione OR calcium OR chromium OR copper OR fluoride OR iodine OR iodide OR iron OR ferrous OR ferric OR magnesium OR manganese OR molybdenum OR phosphorus OR phosphate* OR phosphoric OR potassium OR selenium OR selenomethionine OR sodium OR zinc OR cobalamin OR hydroxycobalamin OR cyanocobalamin OR pyridoxine OR pyridoxal OR pyridoxamine ) |
| --- | --- |
| #2 | TITLE( "stem cell transplant*" OR "stem cell graft*" OR "bone marrow transplant*" OR "bone marrow cell transplant*" OR "bone marrow graft*" OR "hematopoietic transplant*" OR "haematopoietic transplant*" OR "hematopoietic cell transplant*" OR "haematopoietic cell transplant*" OR "graft versus host reaction" OR "graft vs host reaction" OR "graft versus host disease" OR "graft vs host disease" OR gvhd ) |
| #3 | #1 AND #2 |
| #4 | #3 AND ( LIMIT-TO ( DOCTYPE , "ar" ) ) AND ( LIMIT-TO ( LANGUAGE , "English" ) ) |

1. **Web Of Science Search Strategy**

| #1 | antioxidant* OR avitaminosis OR microelement OR "nutrient*" OR "micronutrient*" OR "micro nutrient*" OR "vitamin*" OR "multivitamin*" OR "provitamin*" OR "mineral*" OR "trace element*" OR "supplement*" OR "deficienc*" OR “nutritional deficienc*” OR "carotenoid*" OR "beta carotene" OR "alpha carotene" OR retinol OR "retinal*" OR "retinyl ester*" OR "retinoic" OR thiamin* OR riboflavin OR niacin OR folate OR folic OR "pantothenic acid" OR biotin OR choline OR "ascorbic acid" OR calciferol OR cholecalciferol OR ergocalciferol OR tocopherol OR "alpha tocopherol" OR phytonadion OR phtomenadione OR phylloquinone OR menaquinone OR menadione OR calcium OR chromium OR copper OR fluoride OR iodine OR iodide OR iron OR ferrous OR ferric OR magnesium OR manganese OR molybdenum OR phosphorus OR phosphate* OR phosphoric OR potassium OR selenium OR selenomethionine OR sodium OR zinc OR cobalamin OR hydroxocobalamin OR cyanocobalamin OR pyridoxine OR pyridoxal OR pyridoxamine (Title)  antioxidant* OR avitaminosis OR microelement OR "nutrient*" OR "micronutrient*" OR "micro nutrient*" OR "vitamin*" OR "multivitamin*" OR "provitamin*" OR "mineral*" OR "trace element*" OR "supplement*" OR "deficienc*" OR “nutritional deficienc*” OR "carotenoid*" OR "beta carotene" OR "alpha carotene" OR retinol OR "retinal*" OR "retinyl ester*" OR "retinoic" OR thiamin* OR riboflavin OR niacin OR folate OR folic OR "pantothenic acid" OR biotin OR choline OR "ascorbic acid" OR calciferol OR cholecalciferol OR ergocalciferol OR tocopherol OR "alpha tocopherol" OR phytonadion OR phtomenadione OR phylloquinone OR menaquinone OR menadione OR calcium OR chromium OR copper OR fluoride OR iodine OR iodide OR iron OR ferrous OR ferric OR magnesium OR manganese OR molybdenum OR phosphorus OR phosphate* OR phosphoric OR potassium OR selenium OR selenomethionine OR sodium OR zinc OR cobalamin OR hydroxocobalamin OR cyanocobalamin OR pyridoxine OR pyridoxal OR pyridoxamine (Abstract) |
| --- | --- |
| #2 | "stem cell transplant*" OR "stem cell graft*" OR "bone marrow transplant*" OR "bone marrow cell transplant*" OR "bone marrow graft*" OR "hematopoietic transplant*" OR "haematopoietic transplant*" OR "hematopoietic cell transplant*" OR "haematopoietic cell transplant*" OR "graft versus host reaction" OR "graft vs host reaction" OR "graft versus host disease" OR "graft vs host disease" OR gvhd (Title) |
| #3 | #1 AND #2 |
| #5 | #4 AND (Article (Document Types)) AND (English (Languages)) |

1. **PubMed Search Strategy**

| #1 | “trace elements”[majr:noexp] OR “micronutrients”[majr:noexp] OR “vitamins”[majr:noexp] OR “minerals”[majr:noexp] OR “antioxidants”[majr:noexp] OR “dietary supplements”[majr:noexp] OR “avitaminosis”[majr:noexp] OR “iron deficiencies”[majr:noexp] OR “antioxidant*”[Title/Abstract] OR “avitaminosis”[Title/Abstract] OR microelement[Title/Abstract] OR “nutrient*”[Title/Abstract] OR “micronutrient*”[Title/Abstract] OR “micro nutrient*”[Title/Abstract] OR “vitamin*”[Title/Abstract] OR “multivitamin*”[Title/Abstract] OR “provitamin*”[Title/Abstract] OR “mineral*”[Title/Abstract] OR “trace element*”[Title/Abstract] OR “supplement*”[Title/Abstract] OR “deficienc*”[Title/Abstract] OR “nutritional deficienc*”[Title/Abstract] OR “carotenoid*”[Title/Abstract] OR “beta carotene”[Title/Abstract] OR “alpha carotene”[Title/Abstract] OR retinol[Title/Abstract] OR “retinal*”[Title/Abstract] OR “retinyl ester*”[Title/Abstract] OR “retinoic”[Title/Abstract] OR “thiamin*”[Title/Abstract] OR riboflavin[Title/Abstract] OR niacin[Title/Abstract] OR folate[Title/Abstract] OR folic[Title/Abstract] OR “pantothenic acid”[Title/Abstract] OR biotin[Title/Abstract] OR choline[Title/Abstract] OR “ascorbic acid”[Title/Abstract] OR calciferol[Title/Abstract] OR cholecalciferol[Title/Abstract] OR ergocalciferol[Title/Abstract] OR tocopherol[Title/Abstract] OR “alpha tocopherol”[Title/Abstract] OR phytonadione[Title/Abstract] OR phytomenadione[Title/Abstract] OR phylloquinone[Title/Abstract] OR menaquinone[Title/Abstract] OR menadione[Title/Abstract] OR calcium[Title/Abstract] OR chromium[Title/Abstract] OR copper[Title/Abstract] OR fluoride[Title/Abstract] OR iodine[Title/Abstract] OR iodide[Title/Abstract] OR iron[Title/Abstract] OR ferrous[Title/Abstract] OR ferric[Title/Abstract] OR magnesium[Title/Abstract] OR manganese[Title/Abstract] OR molybdenum[Title/Abstract] OR phosphorus[Title/Abstract] OR “phosphate*”[Title/Abstract] OR phosphoric[Title/Abstract] OR potassium[Title/Abstract] OR selenium[Title/Abstract] OR selenomethionine[Title/Abstract] OR sodium[Title/Abstract] OR zinc[Title/Abstract] OR cobalamin[Title/Abstract] OR hydroxycobalamin[Title/Abstract] OR cyanocobalamin[Title/Abstract] OR pyridoxine[Title/Abstract] OR pyridoxal[Title/Abstract] OR pyridoxamine[Title/Abstract] |
| --- | --- |
| #2 | “Stem Cell Transplantation”[majr:noexp] OR “Bone Marrow Transplantation”[majr:noexp] OR “Graft vs Host Disease”[majr:noexp] OR “stem cell transplant*”[Title] OR “stem cell graft*”[Title] OR “bone marrow transplant*”[Title] OR “bone marrow cell transplant*”[Title] OR “bone marrow graft*”[Title] OR “hematopoietic transplant*”[Title] OR “haematopoietic transplant*”[Title] OR “hematopoietic cell transplant*”[Title] OR “haematopoietic cell transplant*”[Title] OR “graft versus host reaction”[Title] OR “graft vs host reaction”[Title] OR “graft v host reaction”[Title] OR “graft versus host disease”[Title] OR “graft vs host disease”[Title] OR “graft v host disease”[Title] OR gvhd[Title] |
| #3 | #1 AND #2 |
| #4 | #3 NOT (animals [mh] NOT humans [mh]) |
| #5 | Filter: English |

1. **The Academy of Nutrition and Dietetics checklist for Quality Assessment for 16 included studies of the Vitamin and Mineral Requirements for Patients >100 days post-SCT or during GVHD**

|  | Gubrianska et al. [23] | Kananen et al. [26] | Katić et al. [27] | Kerschan-Schindl et al. [28] | Laroche et al. [29] | Ljubas Kelecic et al. [30] | Pereira et al. [32] | Tong et al. [35] | Välimäki et al. [36] | Baumgartner et al. [22] | Hari et al. [24] | Kananen et al. [25] | Kenny et al. [21] | Lu et al. [31] | Stern et al. [33] | Tauchmanovà et al. [34] |
| --- | --- | --- | --- | --- | --- | --- | --- | --- | --- | --- | --- | --- | --- | --- | --- | --- |
| *RELEVANCE QUESTIONS* | Yes | Yes | Yes | Yes | Yes | Yes | Yes | Yes | Yes | Yes | Yes | Yes | Yes | Yes | Yes | Yes |
| *VALIDITY QUESTIONS* |  | | | | | | | | | | | | | | | |
| 1. Was the research question clearly stated? | Yes | Yes | Yes | Yes | Yes | Yes | Yes | Yes | Yes | Yes | Yes | Yes | Yes | Yes | Yes | Yes |
| 2. Was the selection of the study subjects/patients free from bias? | No | No | Yes | No | No | No | No | Yes | No | Yes | No | Yes | No | No | No | Yes |
| 3. Were study groups comparable? | Unclear | Unclear | Unclear | Unclear | Unclear | yes | Unclear | Unclear | No | Yes | Yes | Unclear | No | Yes | No | Yes |
| 4. Was method of handling withdrawals described? | Yes | Yes | Yes | Yes | Yes | Yes | Yes | Yes | Yes | Yes | Unclear | Yes | No | Yes | Yes | Yes |
| 5. Was blinding used to prevent introduction of bias? | No | No | No | No | No | No | No | Yes | No | No | No | No | N/A | No | No | No |
| 6. Were intervention/therapeutic regimens/exposure factor or procedure and any comparison(s) described in detail? Were intervening factors descibed? | Yes | Yes | Yes | Yes | Yes | Yes | Yes | Yes | Yes | Yes | Yes | Yes | Unclear | Yes | Yes | Yes |
| 7. Were outcomes clearly defined and the measurements valid and reliable? | Yes | Yes | Yes | Yes | Yes | Yes | Yes | Yes | Yes | Yes | Yes | Yes | Yes | Yes | Yes | Yes |
| 8. Was the statistical analysis appropriate for the study design and type of outcome indicators? | No | Yes | Yes | Yes | Unclear | Yes | Yes | Unclear | Unclear | Yes | Yes | Unclear | Yes | Yes | No | Yes |
| 9. Are conclusions supported by results with biases and limitations taken into consideration? | Yes | No | Yes | Unclear | No | Unclear | No | Unclear | No | Yes | No | No | No | Yes | No | Yes |
| 10. Is bias due to study's funding or sponsorship unlikely? | Yes | Yes | Yes | Unclear | No | Yes | Unclear | Yes | No | Yes | No | Unclear | Yes | No | Yes | No |
| Quality Rating (-, ∅, +) | ∅ | ∅ | ∅ | ∅ | ∅ | ∅ | ∅ | ∅ | ∅ | ∅ | ∅ | ∅ | ∅ | ∅ | ∅ | ∅ |
| ^b^Positive (+): Indicates that the report has clearly addressed issues of inclusion/exclusion, bias, generalizability, and data collection and analysis | | | | | | | | | | | | | | | | |
| ^c^Negative (-): Indicates that these issues have not been adequately addressed | | | | | | | | | | | | | | | | |
| ^d^Neutral (∅): Indicates that the report is neither exceptionally strong nor exceptionally weak | | | | | | | | | | | | | | | | |
